# Supportive therapies in patients with advanced adrenocortical carcinoma submitted to standard EDP-M regimen

**DOI:** 10.1007/s12020-022-03075-y

**Published:** 2022-05-14

**Authors:** Antonella Turla, Marta Laganà, Salvatore Grisanti, Andrea Abate, Vittorio Domenico Ferrari, Valentina Cremaschi, Sandra Sigala, Francesca Consoli, Deborah Cosentini, Alfredo Berruti

**Affiliations:** 1 Medical Oncology Unit, Department of Medical and Surgical Specialties, Radiological Sciences, and Public Health, University of Brescia, ASST Spedali Civili, Brescia, Italy; 2 Section of Pharmacology, Department of Molecular and Translational Medicine, University of Brescia, Brescia, Italy

## Abstract

**Purpose:**

The management of patients with advanced/metastatic adrenocortical carcinoma (ACC) is challenging, EDP-M (etoposide, doxorubicin, cisplatin combined with mitotane) is the standard regimen. However, it is quite toxic, so an adequate supportive therapy is crucial to reduce as much as possible the side effects and maintain the dose intensity of cytotoxic agents.

**Methods:**

We describe the main side effects of the EDP-M scheme and the best way to manage them based on the experience of the Medical Oncology Unit of the Spedali Civili of Brescia. We also deal with the administration of EDP-M in specific frail patients, such as those with huge disease extent and poor performance status (PS) and those with mild renal insufficiency.

**Results:**

In patients with hormone secreting ACC the rapid control of Cushing syndrome using adrenal steroidogenesis inhibitors such as metyrapone or osilodrostat is mandatory before starting EDP-M. Primary prophylaxis of neutropenia with Granulocyte-Colony Stimulating Factors is crucial and should be introduced at the first chemotherapy cycle. Possible mitotane induced hypoadrenalism should be always considered in case of persistent nausea and vomiting and asthenia in the interval between one cycle to another. In case of poor PS. A 24 h continuous infusion schedule of cisplatin could be an initial option in patients with poor PS as well as to reduce the risk of nefrotoxocity in patients with mild renal impairment.

**Conclusion:**

A careful and accurate supportive care is essential to mitigate EDP-M side effects as much as possible and avoid that, due to toxicity, patients have to reduce doses and or postpone cytotoxic treatment with a negative impact on efficacy of this chemotherapy regimen.

## Introduction

EDP-M (etoposide, doxorubicin, cisplatin combined with mitotane) is the standard first-line regimen in the management of patients with advanced adrenocortical carcinoma (ACC), an extremely rare disease [1, 2]. The efficacy of this regimen is notoriously limited [3, 4], however, it is still the most efficacious regimen we currently have in the management of this disease, leading to complete pathological remission in a small proportion of patients.

Other cytotoxic therapies [5–8], molecular target therapies [9], and immunotherapies [10, 11] administered to patients with disease progression to EDP-M, did not show remarkable efficacy. Based on this premise we should administer the EDP-M regimen in the best possible way in order to obtain the maximum benefit in each patient. Strong evidence shows that cancer patients benefit from the delivery of full-dose cancer chemotherapy. Reducing the delivery of full chemotherapy dose intensity through treatment delays, dose reduction, or early termination of cancer treatment may increase the risk for recurrence and death [12].

EDP-M is quite toxic, but ACC patients are often young and could tolerate full doses. ACC hypersecretion of androgens may promote tolerance to EDP-M although to our knowledge there are no comparative tolerability data in patients stratified by androgen hypersecretion or not, Certainly, maintaining the dose exposes patients to a greater risk of side effects. Moreover, in the EDP-M scheme chemotherapy is administered in association with mitotane, a drug with adrenolytic activity. This association poses problems in interpreting the origin of symptoms and on the best supportive therapy to be administered. In this paper, we will discuss the main side effects of the EDP-M scheme and the best way to manage them based on the experience gained in the Medical Oncology of the Spedali Civili of Brescia, one of the reference centers for ACC in Italy. In addition, we will deal with specific aspects in the management of patients with advanced ACC treated with EDP-M, such as patients with huge disease extent and mild renal insufficiency.

## Management EDP-related toxicities

As previously reported, the most common EDP-M-related toxicities are neutropenia, nausea/vomiting, and asthenia. Cisplatin-related neuropathy also frequently occurs in these patients. The management of these toxicities is necessary to preserve patients’ quality of life and avoid chemotherapy discontinuations.

### Neutropenia

Neutropenia has been reported to occur in 53% of patients at treatment recycle and 77% of patients at nadir [3]. To prevent neutropenia, primary prophylaxis is crucial: Granulocyte-Colony Stimulating Factors should be administered in all patients treated with EDP-M from the first cycle of therapy; G-CSF (Peg-filgrastim) 6 mg/0.6 mL 1 prefilled syringe is usually injected 24–48 h after the end of every cycle. In the management of EDP-M related neutropenia, particular attention should be given to patients with Cushing’s syndrome, which is notoriously associated with infectious diseases and sepsis [13].

In these cases, EDP-related neutropenia is particularly dangerous. Therefore, the rapid control of Cushing syndrome in ACC-eligible EDP-M patients is therefore a priority. Surgery on the primary adrenal mass is undoubtedly a rapid and effective therapeutic procedure to obtain a reduction in circulating levels of cortisol. However, it should be emphasized that debulking surgery of a very aggressive disease can involve a risk of rapid growth of the residual tumor favored by the immunosuppression of the post-surgical period [14]. Furthermore, this therapeutic strategy implies a delay in the initiation of an effective systemic treatment. Several adrenally directed medical therapies are currently available including ketoconazole, metyrapone, osilodrostat, mitotane, and etomidate. These drugs inhibit one or several enzymes involved in adrenal steroidogenesis [15].

Mitotane is highly effective in the long-term management of ACC-induced Cushing’s syndrome and its mechanism of action prevents the escape phenomenon, even after treatment withdrawal, because the drug is stored in adipose tissue and has a long half-life. However, the efficacy of mitotane is often delayed so the association with adrenal steroid inhibitors with a greater rapidity of action is often necessary.

Metyrapone metabolism and elimination are not altered by concomitant mitotane, which is known to be a strong hepatic enzyme inducer and a recent paper has shown that the combination of EDP-M with metyrapone is effective and leads to rapid control of Cushing’s syndrome induced by cortisol-secreting ACC [16]. The addition of ketoconazole to the combination of metyrapone and mitotane may increase its efficacy, as well as osilodrostat can be a useful option in cases where metyrapone is contraindicated or could be not prescribed [17, 18].

Cortisol inhibition after metyrapone and osilodrostat is associated with increased androgen levels, so these drugs are not effective in controlling hyperandrogenism in patients with concomitant cortisol and androgen hypersecretion. For these patients abiraterone, a drug inhibiting 17 alpha hydroxylase and 11–20 lyase, used in the treatment of patients with prostate cancer, could be the most suitable drug as demonstrated by a preclinical in vitro and in vivo experience and confirmed in a published case report. However, Abiraterone is not currently approved in the management of Cushing syndrome [19, 20].

Due to the availability of adrenally directed drugs, ACC patients with Cushing syndrome should be initially treated with these drugs in association with mitotane and EDP regimen should be delayed for 10–15 days when serum cortisol levels are consistently decreased.

### Nausea and vomiting

Nausea and vomiting are extremely frequent in EDP-M treated patients. This side effect was observed in 90% of treated patients. Emesis therefore should be effectively managed both during the EDP-M administration and the following days. Standard antiemetic therapies for cisplatin containing regimen should be administered in patients undergoing EDP-M therapy, following the international guidelines [21]. These includes highly selective serotonin 5-HT(3) receptor antagonists such as palonosetron 0.25 mg intravenously from day 1 to day 4 of chemotherapy infusion; dexamethasone 8 mg/daily from day 1 to day 4. In addition, due to the highly emetogenic effect of EDP-M, aprepitant, a substance P/neurokinin 1 (NK1) receptor antagonist, 125 mg on day 1 and 80 mg on days 2 and 3 of the cycle should be carefully considered. Granisetron transdermal patch is another therapeutic option to be considered for inadequate emesis control between one cycle and the subsequent one. In the experience of the Medical Oncology of Brescia, patients undergoing EDP are at risk of symptoms of hypoadrenalism on the 5th and 6th day when the administration of high doses of dexamethasone is interrupted. These symptoms should be recognized immediately and an extra dose of oral or parenteral glucocorticoids should be given. Our suggestion is that, in ACC patients who are eligible for EDP-M, this therapy should be given preferentially on an inpatient basis in order to better manage supportive care and possible acute hypoadrenalism [22] after cessation of administration of the cytotoxic agents and dexamethasone.

In the period of time between one cycle and the next, particular attention must be given to the management of nausea and, more rarely, vomiting as they may be due to either the effect of cytotoxic drugs or consequent to hypoadrenalism. An extra dose of glucocorticoids should be considered in case of persistent nausea.

### Asthenia

As for nausea, even asthenia is frequently included among EDP-M toxicities occurring in 70% of patients [3]. Also with regard to this symptom, similarly to what previously stated for nausea and vomiting, it is necessary to consider the possibility that it can be due to concomitant hypoadrenalism and extradoses of glucocorticoids should be taken into consideration in case of persistent asthenia.

### Neuropathy

Patients submitted to treatments for advanced ACC often experience neurological toxicities; differentiate neuropathy due to mitotane from the one caused by cisplatin is important, since they are central and peripheral, respectively. Because of the different mechanism of nerval damage, mitotane should not be withdrawn if cisplatin-related peripheral neuropathy occurs during EDP administration.

Until now, no effective agent exists to prevent cisplatin-induced peripheral neuropathy (CIPN). Efficacious pharmacological therapeutic options for patients with established CIPN are limited and CIPN recovery is in general partial with residual deficits in most patients.

## Management of patients with mild renal impairment

Cisplatin is notoriously nephrotoxic and nephrotoxicity is caused by the drug accumulation in renal proximal tubules and typically start approximately 10 days after treatment. In some ACC patients who underwent surgery, there was a need to remove the kidney as it was macroscopically involved. These patients may have mild renal impairment and this can hinder the administration of cisplatin. A nephrologist consultation is crucial before starting cisplatin administration in a patient with renal impairment.

Independent predictors of cisplatin nephrotoxicity are hypoalbuminemia, smoking, female sex, and old age [23].

Consistent hydration (3 L/day) during cisplatin administration associated with sodium bicarbonate infusion and magnesium is recommended for mitigating nephrotoxicity.

Particular attention should be paid on the management of hypoalbuminemia since it leads to an increased concentration of unbound cisplatin which could enhance drug nephrotoxicity [24].

We preferentially hospitalize patients with mild renal impairment who require EDP-M therapy and adopt a 24-hour cisplatin-based infusion on the basis of the results of a study on pediatric cancer patients that reported a less frequent nephrotoxicity with continuous drug administration than with divided doses [25] although the significance of this practice has not been fully established. Many experiences of using carboplatin instead of cisplatin are available in other types of cancers, aiming to benefit through different toxicity profiles [26–29]. However, the use of carboplatin instead of cisplatin is not usually adopted by us as we do not have data on the efficacy and tolerability of EDP with carboplatin in adrenocortical carcinoma.

## Management of patients with poor performance status

Patients with ACC in whom the diagnosis was late, may present with extensive disease and compromised general conditions. In view of the toxicity, EDP-M may not be advisable. In these cases, our strategy is to start with mitotane combined with a single administration of cisplatin, 45 mg/m2, administered in a 24-hour continuous infusion. Previous studies have shown that this administration schedule is well tolerated.

**Fig. 1:**
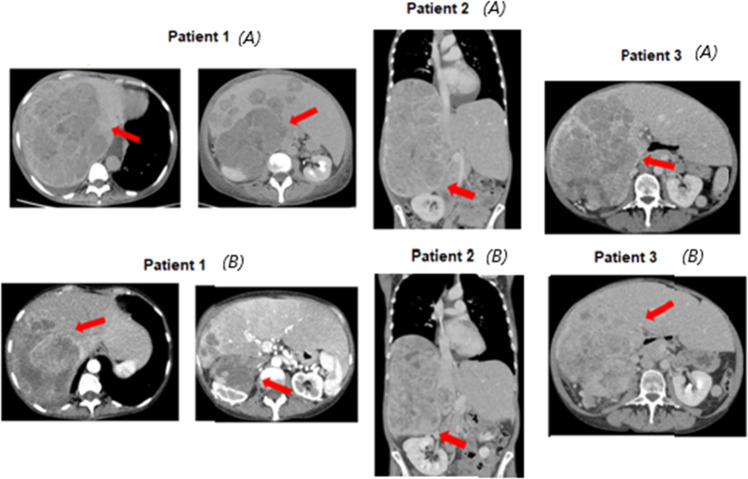
Patients with poor performance status before **A** and after (**B**) chemotherapy (3 cycles) preceded by a single administration of cisplatin (45 mg/m^2^) in a 24-hour continuous infusion

This therapy is also associated with strong nutritional support and drugs that counteract asthenia and improve appetite such as progestins (i.e., megestrol acetate). Patients are subsequently followed up and, if in the following days there is an improvement in their performance status, full EDP doses are administered after 10–15 days. Figure 1 shows the results of this approach in 3 patients with huge disease extent and poor PS who obtained a response to this therapeutic strategy that lasted 8, 7, and 9 months, respectively.

Table 1 shows the toxicity of cisplatin continuous infusion (45 mg/m^2^ daily) in 5 ACC patients in which it was administered for renal insufficiency (2 patients) and poor performance status (3 patients).

**Table 1:** Adverse events related to 24-hour cisplatin in continuous infusion (45 mg/m^2^ daily)

Adverse Events	*N* = 5 patients (%)	
	Any grade	≥Grade 3
Nausea	0	0
Vomiting	0	0
Diarrhea	0	0
Asthenia	2 (40)	0
Constipation	0	0
Hematological toxicity	4 (80)	0
Neutropenia	0	0
Anemia	4 (80)	0
Thrombocytopenia	0	0

We also collected data about five patients affected by ACC and renal disease that underwent standard EDP-M regimen after 24-hour administration (Table 2): as expected the most common chemotherapy-induced toxicities were hematological toxicity (5 patients, 100%), especially anemia (5 patients, 100%), asthenia (3 patients, 60%) and nausea (3 patients, 60%). These side effects, however, were mild to moderate. and, Interestingly, no ≥grade 3 adverse events were reported after the first administration with EDP standard schedule, suggesting that the 24-hour cisplatin administration could increase the patient tolerability of the following EDP. This strategy was successfully adopted in 3 patients (Fig. 1) with huge disease extension and poor PS at presentation who obtained a significant improvement in their general conditions after administration of 24 hour cisplatin infusion plus megestrol acetate and nutritional support. In these patients it was possible to administer EDP-M at full doses after 10–15 days and the results after 3 cycles show disease control in all 3 and an objective response in all of them.

**Table 2:** Adverse events related to 24-hour cisplatin and following EDP-M after first cycle

Adverse events	*N* = 5 patients (%)	
	Any grade	≥Grade 3
Nausea	3 (60)	0
Vomiting	0	0
Diarrhea	0	0
Asthenia	3 (60)	0
Constipation	3 (60)	0
Hematological toxicity	5 (100)	0
Neutropenia	0	0
Anemia	5 (100)	0
Thrombocytopenia	0	0

## Conclusion

In conclusion, EDP-M is an aggressive chemotherapy scheme burdened with significant toxicity. Patients with ACC are however generally young and can tolerate the full doses that must be pursued to achieve the maximum possible efficacy. Supportive therapies aiming to prevent most common chemotherapy toxicities and therapies to rapidly control hormone hypersecretion are crucial. Many recent papers have underlined the benefits obtained combining early supportive therapies with oncologic active therapies for patients with advanced cancer to relieve their symptoms and improve the efficacy of the therapies. The association with mitotane to EDP regimen poses difficulties of interpretation between the typical side effects of chemotherapy (i.e., nausea, vomiting, asthenia) and mitotane-induced hypoadrenalism. ACC patients undergoing mitotane must be carefully followed by a team of expert endocrinologists and oncologists in order to mitigate side effects as much as possible and avoid that, due to toxicity, patients have to reduce doses and or postpone cytotoxic treatment with an impact. negative on efficacy.

In general endocrinologists and oncologists should not have a pessimistic attitude when treating patients with advanced ACC as this leads to the administration of suboptimal treatment delivery. We are aware that the EDP-M scheme has limited efficacy however it can achieve long-lasting disease control in a minority of patients. Such results are not obtained if therapy is inadequately administered for an ineffective management of toxicity.
